# Role of Cysteinyl Leukotrienes in Food Allergy and Anaphylaxis: A Potential Therapeutic Target

**DOI:** 10.1111/all.70393

**Published:** 2026-05-15

**Authors:** Adrienn Weingärtner, Thomas Eiwegger

**Affiliations:** ^1^ Division of Paediatric Pulmonology and Allergology, Department of Paediatrics and Adolescent Medicine Medical University of Graz Graz Austria; ^2^ Karl Landsteiner University of Health Sciences Krems Austria; ^3^ Department of Pediatric and Adolescent Medicine University Hospital St. Pölten St. Pölten Austria; ^4^ Department of Immunology, Temerty Faculty of Medicine University of Toronto Toronto Ontario Canada; ^5^ Translational Medicine, Research Institute Hospital for Sick Children Toronto Ontario Canada

**Keywords:** arachidonate 5‐lipoxygenase, arachidonic acid, cysteinyl leukotrienes, dipeptidase 1, food allergy, gastrointestinal tissue, IgE, immune mechanism, mast cells

Food allergies represent a growing global health concern [1, 2]. Upon allergen exposure, cross‐linking of IgE triggers mast cell degranulation, releasing potent mediators, including histamine, prostaglandins, cytokines, and cysteinyl leukotrienes (CysLTs) [1, 3, 4]. Although the central mediators released by mast cells and basophils are well known, the mechanistic relevance and contribution of leukotrienes in food allergic reactions remain incompletely understood.

In particular, the factors that determine the severity of an allergic reaction remain poorly understood, hindering the identification of high‐risk populations. So far, poorly controlled asthma, age, hereditary alpha‐tryptasemia, mastocytosis, low platelet‐activating factor acetyl hydrolase, and certain drugs have been linked to severe reactions. Extrinsic cofactors may further reduce the threshold for allergic reactions and amplify clinical severity [4].

Recently, two highly relevant publications have emphasized the importance of intestinal mast cells and CysLTs in food allergy [5, 6]. Repeated gastrointestinal exposure to allergens was associated with IgE‐dependent mast cell expansion in the intestinal epithelium, an enrichment of a functionally distinct intestinal epithelial mast cell population with higher CysLT production compared to tissue mast cells in a food allergy model. CysLTs negatively impacted barrier function in the GI tract and were required for anaphylaxis induced by ingestion but not by i.v. injection. This effect was TGF‐beta dependent.

Blocking leukotriene pathways revealed protective effects. Genetic deficiency of Arachidonate 5‐Lipoxygenase (ALOX5), the key enzyme for leukotriene biosynthesis, or of leukotriene C4 synthase (LTC4S) protected mice from anaphylaxis upon intragastric challenge but not in the case of i.v. challenge. This effect was less pronounced upon knockout of leukotriene receptors CysLTR1 or CysLTR2. Pharmacological inhibition mirrored these findings.

Further evidence in this context is provided by Laura R. Hoyt et al. on dipeptidase 1 (DPEP1), an enzyme responsible for degrading leukotriene C4. Mice deficient in Dpep1 displayed elevated CysLT levels and an increased susceptibility to anaphylaxis. A blockade of leukotriene signaling reduced allergen uptake in the gut and prevented systemic reactions. These data reinforce the notion that CysLTs are essential mediators of gastrointestinal anaphylaxis. Thus, genetic and pharmacologic interventions demonstrate that CysLTs exert a pivotal role in food allergy and anaphylaxis. They act through two main mechanisms: (1) driving expansion and activation of intestinal mast cells, and (2) facilitating allergen uptake across the mucosal barrier (Figure 1). By targeting these pathways, the risk and severity of anaphylaxis can be reduced in food allergy models.

**FIGURE 1:**
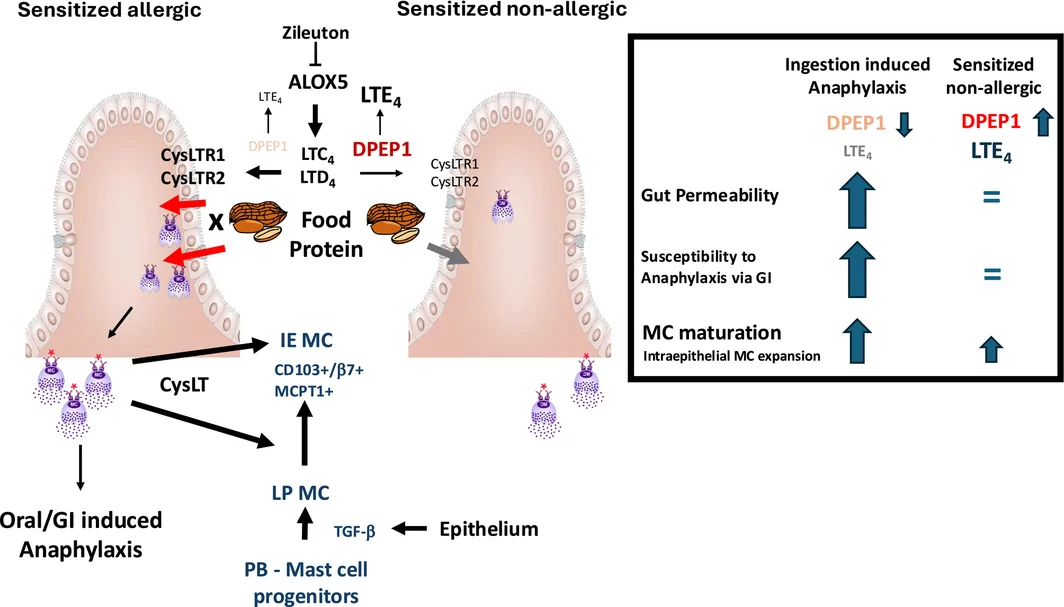
Cysteinyl leukotriene metabolism contributes to ingestion‐induced anaphylaxis. ALOX5 is the rate‐limiting enzyme that is instrumental in initiating the leukotriene biosynthesis. Dipeptidase 1 (DPEP1) drives the conversion of LTD_4_ to LTE_4_. In case of reduced DPEP1 activity, LTD4 accumulates in the gut. Leukotrienes promote intraepithelial mast cell maturation, increased gut permeability and allergen uptake, and susceptibility to gastrointestinal anaphylaxis. ALOX5, Arachidonate 5‐Lipoxygenase; CysLTR, cysteinyl leukotriene receptor; CysLTs, cysteinyl leukotrienes; DPEP1, dipeptidase 1; IE, intra‐epithelial; LP, lamina propria; MC, mast cell; MCPT1, mast cell protease 1.

Importantly, inhibition of the CysLT pathway through ALOX5 blockade was not efficacious in models of intravenous allergen exposure. This indicates that CysLTs contribute only to mucosal sensitization but are dispensable for systemic effector pathways of anaphylaxis in rodent models. The therapeutic window of ALOX5 inhibition was limited, and protection was more pronounced in gastrointestinal rather than systemic allergen challenge models.

Compared with established non‐allergen‐specific treatments like anti‐IgE, which are already approved for the management of food allergies in the US and widely available for other indications, the therapeutic potential of CysLT inhibition needs to be demonstrated. This is particularly relevant in the context of more affordable anti‐IgE generics now available. It is tempting to speculate that drugs such as zileuton, an ALOX5 inhibitor, may also provide benefit in selected scenarios, such as secondary allergy prevention or risk reduction, and for side‐effect prevention in the context of OIT. This would further expand the toolbox of anti‐allergic treatment options for patients with food allergy. Thus, while targeting CysLTs offers an attractive mechanistic approach, its clinical application in food allergy may be confined to specific patient groups or used as adjunctive therapy. Moreover, ALOX5 inhibition may affect lipid metabolism at different levels.

Taken together, these two studies reveal that CysLTs produced by a distinct subpopulation of intestinal mast cells critically modulate barrier function during sensitization and drive the anaphylactic response to ingested antigens. This opens the possibility of novel options to prevent and treat food‐induced anaphylaxis.

## Funding

This study was supported by the Luxembourg National Research Fund on WEAVE program grant C23/BM/18096599/IFAM, the Austria Science Fund on WEAVE program grant https://doi.org/10.55776/I6897, and the lower Austrian research fund supported Danube Allergy Research Cluster.

## Conflicts of Interest

The authors declare no conflicts of interest.

## Data Availability

The authors have nothing to report.
